# Paths to improving engagement among racial and ethnic minorities in addiction health services

**DOI:** 10.1186/s13011-015-0036-z

**Published:** 2015-10-26

**Authors:** Erick G. Guerrero, Karissa Fenwick, Yinfei Kong, Christine Grella, Thomas D’Aunno

**Affiliations:** School of Social Work, University of Southern California, 655 West 34th Street, Los Angeles, CA 90089 USA; Department of Psychiatry & Biobehavioral Sciences, University of California, Los Angeles, Integrated Substance Abuse Programs, Los Angeles, USA; Wagner Graduate School of Public Service, New York University, New York, USA

**Keywords:** Program capacity, Comprehensive care, Racial and ethnic minorities, Wait time, Retention

## Abstract

**Background:**

Members of racial and ethnic minority groups are most likely to experience limited access and poor engagement in addiction treatment. Research has been limited on the role of program capacity and delivery of comprehensive care in improving access and retention among minorities with drug abuse issues. The goal of this study was to examine the extent to which access and retention are enhanced when racial and ethnic minorities receive care from high-capacity addiction health services (AHS) programs and via coordination with mental health and receipt of HIV testing services.

**Methods:**

This multilevel cross-sectional analysis involved data from 108 programs merged with client data from 2011 for 13,478 adults entering AHS. Multilevel negative binomial regression models were used to test interactions and indirect relationships between program capacity and days to enter treatment (wait time) and days in treatment (retention).

**Results:**

Compared to low-capacity programs and non-Latino and non-African American clients, Latinos and African Americans served in high-capacity programs reported shorter wait times to admission, as hypothesized. African Americans also had longer treatment retention in high-capacity programs. Receipt of HIV testing and program coordination of mental health services played an indirect role in the relationship between program capacity and wait time.

**Conclusions:**

Program capacity and coordinated services in AHS may reduce disparities in access to care. Implications for supporting low-capacity programs to eliminate the disparity gap in access to care are discussed.

## Background

Addiction health services (AHS) programs face significant challenges to develop capacity to expand service delivery and enhance engagement of clients in treatment [1]. The Affordable Care Act provides an opportunity to expand AHS capacity by increasing access to public health insurance and encouraging service integration [2]. This is particularly critical for underserved populations that face numerous barriers to accessing AHS, resulting in disparities in access to needed services [3]. Therefore, it is critical to understand program factors that may facilitate the delivery of coordinated care services in AHS to reduce disparities in access and engagement among low-income and racial and ethnic minority populations [3–5]. Building on a recent study suggesting that high program capacity helps reduce client wait time and increase retention in AHS programs [6], the current study examined the extent to which access and retention are enhanced when racial and ethnic minorities receive care from high-capacity AHS programs and via coordination with mental health providers and receipt of HIV testing services.

Among individuals seeking help for substance abuse issues, waiting to enter treatment is the most commonly cited barrier [7–9], and treatment access and retention are critical predictors of reduced posttreatment substance use [10, 11]. Exploring program factors, such as program capacity, that enhance client wait time and retention in AHS is critical because these outcomes are important predictors of treatment success and increasingly used in program evaluation [5, 6, 12, 13]. Although program capacity is frequently assessed using measures of organizational size (such as number of clients served or number of staff members), we developed a more comprehensive measure of program capacity that reflects the necessary components (i.e., funding, workforce and infrastructure, and leadership) to thrive in the current health care service context. We defined high-capacity AHS programs as those with high directorial leadership capacity, organizational readiness for change (ORC), and capacity to bill Medicaid [6].

Leadership is essential to fostering change, developing resources, and improving performance [14], particularly directorial leadership that is transactional (relies on incentives) and transformational (promotes human development) [15]. Leadership influences the implementation of evidence-based practices and provision of integrated services [16, 17], and leadership behavior is associated with client wait time [18]. ORC, a widely used construct of AHS program functioning, is associated with effective implementation of new practices and innovations [19]. In addition, clients in programs with high levels of ORC report greater treatment rapport, satisfaction, and participation [20]. Finally, in this era of Medicaid expansion, acceptance of Medicaid provides revenue that allows programs to support more services, comply with quality expectations from funders, and enhance access for low-income individuals [1–3, 6]. In previous work, we demonstrated how a latent class variable using leadership, ORC, and Medicaid acceptance can serve as a proxy for program capacity and distinguish between high- and low-capacity programs based on client outcomes. In this study, we used this latent program capacity variable to efficiently examine the extent to which disparities in wait time and retention are reduced when racial and ethnic minorities receive treatment from high-capacity programs and via coordination with mental health and receipt of HIV testing services.

Racial and ethnic minorities such as African Americans and Latinos often fare worse than their White counterparts in terms of wait time and retention [3, 21]. Emerging research has suggested that treatment disparities exist partly because racial and ethnic minority clients are more likely to access low-capacity programs that are not able to meet their service needs [6, 22]. However, high-capacity programs have the resources to facilitate greater knowledge of and connections with their communities and are more responsive to the needs of the minority populations they serve. Therefore, they may be able to tailor outreach and engagement to Latino and African American clients, enhancing wait time and retention for these clients beyond that of Whites. Hence, we tested whether receiving care from a high-capacity program that has strong leadership, readiness to adopt new practices, and acceptance of public insurance eliminates differences among racial and ethnic groups in key measures of treatment engagement. We posited in Hypothesis 1 that African American and Latino clients accessing high-capacity AHS programs would have (a) lower wait time and (b) increased retention compared to Whites.

Coordination of care can help reduce disparities and improve treatment outcomes [22]. AHS coordination with services such as mental health treatment and health counseling is associated with improved client retention in treatment and posttreatment substance use [23]. The Affordable Care Act and Mental Health Parity and Addiction Equity Act provide incentives for coordination of care by requiring health insurance plans to offer mental health and substance abuse treatment at the same level as medical benefits [1, 24]. As more individuals enter treatment, organizations will likely seek partnerships to accommodate the demand. Because mental health and HIV testing services are two of the most needed services for clients in publicly funded AHS programs [5, 22, 25] and previous work has shown that components of high-capacity programs are associated with coordination of mental health and HIV testing services [26], we examined the extent to which coordinated care is the mechanism by which program capacity is associated with wait time and retention. We posited in Hypothesis 2 that high-capacity programs would be indirectly associated with (a) reduced client wait time and (b) increased client retention through coordination of mental health and receipt of HIV testing services.

## Methods

### Sampling frame

This study used a fully concatenated program and client dataset collected in 2010 and 2011. The sampling procedures for these data have been described in detail elsewhere [6]. All study procedures were approved by the University of Southern California Institutional Review Board. The analytic sample for the current study consisted of 108 AHS programs and 13,478 client treatment episodes drawn from programs located in communities with a population of 40 % or more African American or Latino residents or both in Los Angeles County. Program-level data were collected from a clinical supervisor at each agency via electronic surveys, with informed consent obtained prior to beginning the survey. Site visits to review program records or collect additional data used to cross-validate program-level survey data were conducted with 91 % of programs. Client data were collected as part of the Los Angeles County Participant Reporting System, the countywide administrative data system required of publicly funded programs, and electronically recorded by clinicians at admission and discharge.

### Measures

Dependent variables were wait time and retention. Wait time was measured as the number of days each client reported spending on a waiting list prior to starting treatment. Retention was measured as the number of days between admission and discharge dates as reported by program staff members in the Los Angeles County Participant Reporting System. These measures are widely used in addiction services research and have been previously demonstrated to be predictive of positive treatment outcomes [5, 11, 27]. Table 1 features the response format and Cronbach’s alpha values of study measures, including a list of independent and control variables.

**Table 1:** Program (*N* = 108) and client (*N* = 13,478) variables in addiction health services

	Mean ± Standard Deviation or n (%)	Response format
Dependent variables		
Wait time	3.36 ± 16.63	Time to enter treatment (days)
Retention	90.46 ± 99.72	Treatment duration (days)
Independent variables		
Program capacity		
High program capacity	8 (7.4)	Latent class (high leadership, readiness for change, Medi-Cal acceptance)
Directorial leadership	40.11 ± 7.04	9 items, scale from 10 (*low*) to 50 (*high*); e.g., Your director inspires others with plans for the facility’s future; α = .96
Readiness for change	34.53 ± 2.80	4 subscales, scale from 10 (*low*) to 50 (*high*).
		Motivational readiness^a^: 24 items, 30.78 ± 5.68, e.g., Your program needs more training for effective implementation of EBPs; α = .80
		Resources: 12 items, 37.94 ± 5.15, e.g., Clinical management decisions for your program are well planned; α = .74
		Staff attributes: 8 items, 40.30 ± 4.01, e.g. You are able to adapt quickly when you have to make changes; α = .86
		Organizational climate: 16 items, 34.66 ± 4.90, e.g. You feel encouraged to try new and different techniques; α = .78
Medi-Cal acceptance	81 (75)	Program accepts Medi-Cal payment
Mental health coordination	45 (42)	On-site and off-site coordination with mental health services
Receipt of HIV testing services^b^	8,165 (74)	Number of clients who received on-site and off-site HIV testing services while in the program
Client race and ethnicity		
African American	2,731 (20)	Self-identify as African American
Latino	5,010 (37)	Self-identify as Latino
White	4,179 (31)	Self-identify as White (non-Latino)
Other	1,558 (12)	Self-identify as other race or ethnicity
Control variables		
Program characteristics		
Public funding^a^	34.2 ± 43.0	Percentage of public funding during previous fiscal year
License	103 (96.3)	Licensed by state
Accreditation	16 (15.2)	Accredited by the Joint Commission
Program type		
Outpatient	60 (55.6)	Primarily outpatient services
Methadone	4 (3.7)	Primarily methadone maintenance services
Residential	36 (33.3)	Primary residential services
Client characteristics		
Referral source^b^		
Self	5,780 (43)	Self-referred
Community	2,262 (17)	Referred by a community organization
Proposition 36	1,896 (14)	Referred by court in lieu of incarceration
Drug court	755 (6)	Referred by drug court
Social services	2,785 (21)	Referred by social services or county agency
Medi-Cal eligible	5,350 (40)	Eligible for Medi-Cal
Mental health issues	3,205 (24)	Diagnosed with mental health issue
Homeless	2,296 (17)	Unstable housing status

^a^More than 8 % data missing

^b^Client-reported characteristic

Our independent variables were program capacity and coordination of services. Measure development is described in detail elsewhere [13]. Program capacity was measured by a latent class variable using the following three variables: (a) leadership, (b) ORC, and (c) acceptance of Medi-Cal payments (California’s Medicaid program). We measured leadership using the Multifactor Leadership Questionnaire [15]. Clinical supervisors rated their director’s leadership on a 5-point scale (1 = *strongly disagree* to 5 = *strongly agree*), and scores were totaled as recommended. We used the Organizational Readiness for Change Scale to measure program readiness to implement new practices. ORC was measured with 67 items divided into four domains with 18 subscales: motivational readiness (three subscales: program and training needs, and pressure for change); resources (five subscales: offices, staffing, training resources, equipment, and Internet access); staff attributes (four subscales: growth, efficacy, influence, and adaptability); and organizational climate (six subscales; mission, cohesion, autonomy, communication, and change) [19, 28]. Items were rated on a 5-point Likert scale (1 = *strongly disagree* to 5 = *strongly agree*). Items from subscales were added and averaged to create scores for each of the four domains, which in turn were averaged and multiplied by 10 to develop the ORC total mean score; higher scores indicated greater readiness. Item examples and alpha levels are provided for all subscales in Table 1. We also measured acceptance of Medi-Cal with a single item asking whether providers had the capacity to accept Medi-Cal payments.

To develop the latent class variable, we relied on latent profile analysis to identify levels of program capacity. Latent profile analysis can incorporate continuous, ordinal, and categorical indicators, in contrast to latent class analysis, which can only accommodate categorical indicators. We determined latent classes that represented different levels of program capacity by considering different solutions for multiple latent profiles (e.g., two classes, three classes, etc.). The full procedure is described in a previous study [6]. The fundamental equation [29] of the latent profile model (Equation 1) was expressed as:

$$ {\sigma}_{ij}^2={\displaystyle \sum_{k=1}^K}{\pi}_k{\left({\mu}_{ik}-{\mu}_i\right)}^2+{\displaystyle \sum_{k=1}^K}{\pi}_k{\sigma}_{ijk}^2 $$

In Equation 1, *i* and *j* (*i* ≠ *j*) are index-specific variables and *k* designates a specific latent class, such that ${\mu}_{ik}$ represents the mean and ${\sigma}_{ijk}^2$ represents the variance for variable *i* in group *j*, *k* is the total number of latent classes, and ${\pi}_k$ indicates the proportion of cases belonging to each class ($\sum_{k=1}^K{\pi}_k=1$). We selected two as the appropriate number of latent classes after testing several models. These two classes represented high- and low-capacity programs, categories represented in the field as small recovery programs and large health care providers [1, 5]. To create two interactions using program capacity and race and ethnicity, we first developed mutually exclusive categories of race (non-Latino White and non-Latino African American), and ethnicity (non-White and non-African American Latino). The interactions included a dichotomous measure of program capacity (high vs. low) and race (African American vs. non-African American) and program capacity (high vs. low) and ethnicity (Latino vs. non-Latino).

To assess coordination with mental health providers, supervisors rated how frequently their program collaborated with mental health and psychiatric providers to coordinate care for clients with dual disorders (1 = *never* to 5 = *always*). This measure resulted in bimodal distributions in the never, almost never, and always categories; thus, we transformed it to a dichotomous measure representing high coordination with mental health providers. To measure receipt of HIV testing services during treatment, clients reported at discharge whether they received HIV testing on- or off-site while in treatment.

Variables had less than 8 % missing data, with the exception of the motivational readiness subscale of the ORC scale (13.97 %) and public funding (8.56 %). We handled missing data using multiple imputation, in which each missing value was replaced with 20 plausible values using the Markov Chain Monte Carlo method [30]. We imputed program and client variables separately using fully conditional specification for multivariate imputation [31]. We developed, merged, and analyzed the 20 imputed datasets using Stata’s MI IMPUTE and MI ESTIMATE commands.

### Statistical analysis

We used multilevel negative binomial regression with robust standard errors in Stata for our analyses, using MI ESTIMATE: NBREG with a log link function [32, 33). The CLUSTER option was used to account for the multilevel structure of the data (clients nested in programs) and obtain more accurate estimates of standard errors [34]. The negative binomial approach was used because the dependent variables were overdispersed count measures of number of days (i.e., their variance was much greater than their mean). Hence, results are expressed as incidence rate ratios (IRR), interpreted as the estimated rate ratio for a 1-unit increase in the independent variable, given the other variables are held constant in the model. To test our hypotheses regarding whether the relationship between program capacity and client wait time (Hypothesis 1a) and retention (Hypothesis 1b) differed by client race and ethnicity, we conducted multilevel linear modeling [32]. To test our hypotheses regarding the indirect relationship between program capacity and client wait time (Hypothesis 2a) and retention (Hypothesis 2b) via coordination with mental health and receipt of HIV prevention services, we used path analysis in R package mediation with a bootstrap *p*-value specification [35].

## Results

**Fig. 1:**
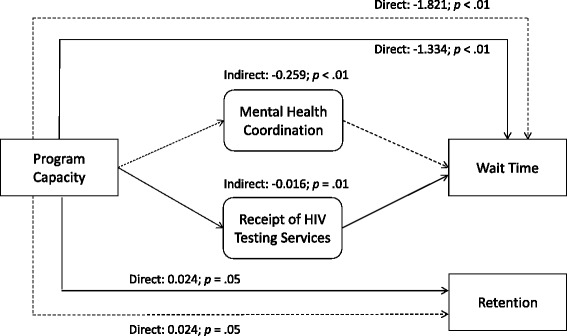
Program capacity, service coordination, and wait time and retention among minorities. Note: Only significant indirect paths are reported. *P*-values represent bootstrap *p*-values. Dotted lines represent estimates including mental health coordination indirect path; solid lines represent path estimates including receipt of HIV testing services indirect path. White is the reference category for race and ethnicity; low program capacity is the reference category for high program capacity. The analytic sample is *N* = 13,478. We adjusted for program-level variables (private insurance, organizational cultural competence, and public funding) and client-level variables (gender, mental illness, homelessness, Medi-Cal eligibility, referral type, race, treatment type)

Descriptive statistics for all variables are shown Table 1. Hypothesis testing results are shown in Table 2 and Fig. 1 and account for all control variables. There were only eight programs (7 %) with high capacity in leadership, high ORC, and acceptance of Medi-Cal payments; 42 % of programs had high coordination with mental health providers and 48 % facilitated HIV testing services on- or off-site to 74 % of clients who received this service during treatment. The average client wait time was 3 days, whereas the average duration in treatment was 90 days. Latinos represented 37 % of the client population, whereas African Americans represented 20 %.

**Table 2:** Multi-level negative binomial models of wait time and retention (*N =* 13,478)

	Wait time		Retention	
	IRR	95 % CI	IRR	95 % CI
Program capacity				
Latent variable components				
Medi-Cal acceptance	0.564***	0.489, 0.650	1.041	0.975, 1.112
Readiness for change	0.705***	0.679, 0.732	1.014*	1.001, 1.026
Directorial leadership	1.004	0.991, 1.016	1.000	0.994, 1.006
Program capacity × Latino^a^	0.131***	0.065, 0.267	1.118	0.950, 1.317
Program capacity × African American^a^	0.237***	0.170, 0.332	1.315***	1.170, 1.479
Organizational characteristics				
Cultural competence	1.021	0.997, 1.047	1.007	0.997, 1.017
Mental health coordination	1.116	0.919, 1.354	0.970	0.904, 1.040
Public funding	0.995***	0.993, 0.997	1.000	0.999, 1.001
Program type^b^				
Methadone	0.381*	0.180, 0.804	0.834**	0.738, 0.942
Residential	4.011***	3.517, 4.576	0.588***	0.561, 0.616
Client characteristics				
Receipt of HIV testing services	1.300***	1.197, 1.413	0.973	0.943, 1.005
Referral source^c^				
Community	0.839*	0.712, 0.988	1.024	0.975, 1.074
Proposition 36	1.560***	1.376, 1.770	0.897***	0.848, 0.948
Drug court	0.992	0.835, 1.177	1.209***	1.133, 1.290
Social service	1.042	0.952, 1.140	0.921***	0.882, 0.962
Medi-Cal eligible	0.990	0.893, 1.098	1.132***	1.084, 1.182
Race and ethnicity^d^				
African American	7.610***	3.636, 15.928	0.952	0.797, 1.136
Latino	4.558***	3.195, 6.503	0.787***	0.692, 0.896
Other	0.766**	0.632, 0.928	1.067	0.997, 1.141
History of mental health	0.907*	0.828, 0.994	0.976	0.941, 1.012
Homeless	1.194***	1.097, 1.300	1.065**	1.021, 1.112

*CI* confidence interval; *IRR* incidence rate ratio. IRRs can be interpreted as the estimated rate ratio for a 1-unit increase in the independent variable, given the other variables are held constant in the model. For example, compared to programs that do not accept Medi-Cal, programs accepting Medi-Cal are associated with a decreased ratio of number of days waiting of IRR = 0.564, while holding all other variables in the model constant. Model statistics for wait time: *F*(21, 9199.7) = 80.20, *p*-value of *F* < 1 = .0001. Model statistics for retention: *F*(21, 40245.1) = 49.9, *p*-value of *F* < 1 = .0001. The corresponding *p*-value is less than .001

^a^Low program capacity and non-Latino White are reference categories

^b^Outpatient is reference category

^c^Self-referral is reference category

^d^Non-Latino White is reference category

*p < .05, **p < .01, ***p < .001

We found support for Hypothesis 1a, which posited that the relationship between high-capacity AHS programs and client wait time would be moderated by client minority status. Latino (IRR = 0.131; 95 % CI = 0.065, 0.267; *p* < .001) and African American (IRR = 0.237; 95 % CI = 0.170, 0.332; *p* < .001) clients entering high-capacity programs had significantly shorter wait times compared to Whites.

We found partial support for Hypothesis 1b, which stated that the relationship between high-capacity programs and client retention in treatment would be moderated by client minority status. African American clients (IRR = 1.315; 95 % CI = 1.170, 1.479; *p* < .001) had significantly greater retention in treatment than White clients. However, Latino clients were not associated with greater retention in treatment at a statistically significant level (*p* > .05).

We found support for Hypothesis 2a, which posited that program capacity would be indirectly associated with client wait time through coordination of care. Program capacity was indirectly associated with client wait time through both coordination with mental health services (standardized indirect effect = −.259, bootstrap *p* < .01) and receipt of HIV testing services (standardized indirect effect = −.016, bootstrap *p* < .01). We did not find support for Hypothesis 2b regarding the relationship among coordination of care, receipt of HIV testing services, and client retention.

Two components of program capacity (Medi-Cal acceptance and ORC) were negatively associated with wait time, whereas only ORC was associated with higher retention (IRR = 1.014; 95 % CI = 1.001, 1.026; *p* = .04). Receipt of HIV testing services was positively associated with wait time (IRR = 1.300; 95 % CI = 1.197, 1.413; *p* < .001). Among control variables, public funding (IRR = 0.995; 95 % CI = 0.993, 0.997; *p* < .001), methadone (IRR = 0.381; 95 % CI = 0.180, 0.804; *p* < .05), and community referrals (IRR = 0.839; 95 % CI = 0.712, 0.988; *p* < .05) were negatively associated with wait time, whereas drug court referral (IRR = 1.209; 95 % CI = 1.133, 1.290; *p* < .001), Medi-Cal eligibility (IRR = 1.132; 95 % CI = 1.084, 1.182; *p* < .001), and homelessness (IRR = 1.065; 95 % CI = 1.021, 1.112; *p* < .01) were positively associated with retention.

## Discussion

This paper examined the extent to which access and retention are enhanced when racial and ethnic minorities receive care from high-capacity AHS programs and via coordination with mental health providers and receipt of HIV testing services. Findings demonstrate how program capacity, as measured in this study, can be used to further examine pathways and interacting relationships that may improve access to care (i.e., wait time). Race and ethnicity and coordinated mental health and receipt of HIV testing services played an important role in reducing wait time. Path analysis showed that high-capacity programs are associated with lower wait time for all clients through coordination with mental health providers and receipt of HIV testing services. Albeit conjectural, it may be that high-capacity programs situated in communities with large percentages of racial and ethnic minority groups are better able to address specific barriers to treatment and provide more rapid treatment entry by connecting with mental health and HIV testing providers. Programs that understand their community’s service needs may become more responsive to clients’ treatment access needs.

Findings also showed that when African American clients entered high-capacity programs, they experienced longer treatment retention than Whites and clients entering low-capacity programs. These results suggest that high-capacity programs are better able to engage African Americans, potentially providing care that aligns better with their needs. Engaging clients in treatment is certainly a complex issue. Although we accounted for client and program factors that play a role in retention, other factors were not included in this study, such as a client’s psychological readiness to engage in treatment and receipt of needed services [23].

However, our latent concept of program capacity allowed us to test a conceptual framework of capacity and follow an efficient methodological approach to compare programs using a multilevel path analysis with complex outcomes (number of days to enter and stay in treatment). This approach is needed to compare programs in a large system of care, such as the AHS system in Los Angeles County, with the purpose of informing system-level interventions. However, understanding the role of different components of program capacity (leadership, ORC, and Medi-Cal payment acceptance) in wait time and retention is also critical, particularly to inform program-level interventions. Results provided in Table 2 show that two capacity factors (ORC and Medi-Cal payment acceptance) were associated with lower wait time, whereas only ORC was associated with higher retention in treatment. These findings support the notion that access to care for low-income populations may be driven by public funding and regulatory and program practices and resources, and that these practices and clients’ referral from criminal justice and eligibility for Medi-Cal also play an important role in retention.

Another important contribution of this paper is its preliminary evidence suggesting that coordination with mental health providers and receipt of HIV testing services enable high-capacity programs to reduce client wait times. High-capacity programs seem to have more resources in terms of infrastructure, workforce training, leadership, and funding. Through their network connections with other providers offering needed services, these high-capacity programs may be able to get clients into treatment faster. However, coordination of specific services, such as mental health treatment or HIV testing, did not play a role in the relationship between program capacity and retention. To address the possibility that our measure of retention did not accurately reflect successful engagement, we conducted post hoc analyses with completion of the treatment episode as the outcome. These results also showed a statistically nonsignificant path through coordination of services in the relationship between program capacity and treatment completion. One explanation for these findings may be that our measurements of mental health coordination at the program level did not allow us to assess whether clients most in need of mental health services actually received these services, thus supporting successful completion of their treatment episode. Ensuring receipt of needed services seems critical in engagement; previous research has demonstrated improved retention based on matching services with client needs [23]. In addition, it is likely that client characteristics, such as drug use severity and socioeconomic status, play a more salient role in treatment retention than program factors such as coordination of services [22].

Limitations associated with study data must be considered when interpreting findings. First, although our analysis examined direct and indirect relationships, we did not extend our results to causal or temporal relationships given that our data were cross-sectional. Our path analysis approach was informed by our conceptual framework described in the introduction. Second, program measures were provided by one manager per program and may have been influenced by social desirability bias. We attempted to reduce this bias by completing validity checks with 91 % of the sample during site visits. This procedure is described elsewhere [36]. Nonetheless, our results are robust because of our use of large multilevel data on clients and programs and reliance on a representative sample of programs serving urban communities in Los Angeles County with more than 7 million residents.

## Conclusions

Results highlight the importance of measuring program capacity in the rapidly changing service delivery environment in the current health care reform context. Findings may inform system and program interventions. Programs can be distinguished based on their capacity—for example, low-capacity programs can be quickly targeted with system interventions to improve client outcomes throughout the county. As a program-level intervention, it is critical to invest in leadership training, staff readiness for change, and Medi-Cal payment acceptance and reporting capacity to improve the quality of care provided to underserved clients. Although current federal programs sponsored by the Substance Abuse and Mental Health Services Administration on leadership development [37], evidence-based practice training, Medicaid payment acceptance, and integration of care [38, 39] are available, these programs may not be sufficient to reach low-capacity community-based treatment programs. These programs need significant direct support from their local government and service networks to build capacity in the components reported here to have the potential to eliminate the current disparity gap in access to care.

### Ethics, consent and permissions

This study was reviewed and approved by the Institutional Review Board of the University of Southern California. The principal investigator, also the corresponding author, has obtained consent to publish from the participants in this study (treatment clients and program staff members).
